# Alteration of Neural Network Activity With Aging Focusing on Temporal Complexity and Functional Connectivity Within Electroencephalography

**DOI:** 10.3389/fnagi.2022.793298

**Published:** 2022-02-04

**Authors:** Momo Ando, Sou Nobukawa, Mitsuru Kikuchi, Tetsuya Takahashi

**Affiliations:** ^1^Graduate School of Information and Computer Science, Chiba Institute of Technology, Narashino, Japan; ^2^Department of Computer Science, Chiba Institute of Technology, Narashino, Japan; ^3^Department of Preventive Intervention for Psychiatric Disorders, National Institute of Mental Health, National Center of Neurology and Psychiatry, Tokyo, Japan; ^4^Department of Psychiatry and Behavioral Science, Kanazawa University, Ishikawa, Japan; ^5^Research Center for Child Mental Development, Kanazawa University, Kanazawa, Japan; ^6^Department of Neuropsychiatry, University of Fukui, Yoshida, Japan; ^7^Uozu Shinkei Sanatorium, Uozu, Japan

**Keywords:** EEG signal, multi-scale entropy, aging, functional connectivity, multi-fracial

## Abstract

With the aging process, brain functions, such as attention, memory, and cognitive functions, degrade over time. In a super-aging society, the alteration of neural activity owing to aging is considered crucial for interventions for the prevention of brain dysfunction. The complexity of temporal neural fluctuations with temporal scale dependency plays an important role in optimal brain information processing, such as perception and thinking. Complexity analysis is a useful approach for detecting cortical alteration in healthy individuals, as well as in pathological conditions, such as senile psychiatric disorders, resulting in changes in neural activity interactions among a wide range of brain regions. Multi-fractal (MF) and multi-scale entropy (MSE) analyses are known methods for capturing the complexity of temporal scale dependency of neural activity in the brain. MF and MSE analyses exhibit high accuracy in detecting changes in neural activity and are superior with regard to complexity detection when compared with other methods. In addition to complex temporal fluctuations, functional connectivity reflects the integration of information of brain processes in each region, described as mutual interactions of neural activity among brain regions. Thus, we hypothesized that the complementary relationship between functional connectivity and complexity could improve the ability to detect the alteration of spatiotemporal patterns observed on electroencephalography (EEG) with respect to aging. To prove this hypothesis, this study investigated the relationship between the complexity of neural activity and functional connectivity in aging based on EEG findings. Concretely, MF and MSE analyses were performed to evaluate the temporal complexity profiles, and phase lag index analyses assessing the unique profile of functional connectivity were performed based on the EEGs conducted for young and older participants. Subsequently, these profiles were combined through machine learning. We found that the complementary relationship between complexity and functional connectivity improves the classification accuracy among aging participants. Thus, the outcome of this study could be beneficial in formulating interventions for the prevention of age-related brain dysfunction.

## 1. Introduction

Complex temporal variability within brain activity plays an important role in perceptual and overall mind and behavioral processes and is known to be a mechanism for stochastic resonance and facilitation (as reviewed in McDonnell and Ward, 2011; Garrett et al., 2013; Takahashi, 2013; Yang and Tsai, 2013; Nobukawa and Nishimura, 2020). Moreover, various memory function components, cognitive functions, and perceptual functions of the brain are associated with brain activity at each temporal scale, as well as with frequency-band specific behaviors, such as theta, beta, alpha, and gamma bands (Klimesch et al., 2007). Therefore, studies using high time resolution for electroencephalography (EEG) and magnetoencephalography (MEG) are currently being conducted to evaluate the complexity of high-frequency components. In particular, neural fluctuations with temporal scale dependency, which can be observed with EEG and MEG, including their relationship with cognitive function (McIntosh et al., 2008), development (Hasegawa et al., 2018), aging (Takahashi et al., 2009, 2016; Nobukawa et al., 2019a), and the pathology of mental disorders (Takahashi et al., 2010; Ahmadlou et al., 2011; Nobukawa et al., 2019b, 2020a), have been extensively studied. Multi-scale entropy (MSE) and multi-fractal (MF) analyses are widely utilized as an effective evaluation method for complexity with temporal scale dependency (as reviewed in Takahashi, 2013; Yang and Tsai, 2013). These methods that focus on the temporal scale dependency of complexity can capture alterations in brain activity within a variety of psychiatric disorders (Yang and Tsai, 2013). In particular, EEG signals in schizophrenia have been reported to be less complex in the frontal region based on a slow temporal scale (Paulus et al., 1996; Takahashi et al., 2010). Similarly, patients with bipolar disorder show less neural complexity (Gottschalk et al., 1995). Considering the temporal scale dependence within EEG for Alzheimer's disease (AD), MSE analysis showed low complexity in the frontal region (Mizuno et al., 2010; Ni et al., 2016). In addition, MF analysis can detect the severity of cognitive impairment in AD (Zorick et al., 2020). Moreover, recent studies have shown that the profile of temporal complexity for EEG signals can be utilized for classifying EEG for AD, and the combination of complexity profiles obtained via MF and MSE enhances the accuracy of AD identification based on their complementary relationship (Zorick et al., 2020; Ando et al., 2021). Consequently, approaches for combining complexity profiles could open new avenues for the identification and characterization of the complex patterns of neural activity regarding cognitive alteration in psychiatric disorders.

In addition to the complex temporal variability, functional connectivity reflects the integration of brain information processes in each neural region, which are represented as mutual interactions of neural activity among brain regions (reviewed in Varela et al., 2001; Buzsáki and Draguhn, 2004; Fries, 2005; Hutchison et al., 2013). Therefore, functional connectivity correlates with cognitive function and alters several pathological conditions characterized by impairments in cognitive function, such as AD (Hata et al., 2016; Yu et al., 2016), autism spectrum disorder (ASD) (Righi et al., 2014), and attention deficit hyperactivity disorder (ADHD) (Ueda et al., 2020). Functional connectivity reflected in EEG has been quantified by coherence, correlation, and mutual information analyses, which reflect the degree of synchronization of neural activity between brain regions (Aertsen et al., 1989; Friston et al., 1993; Bullmore and Sporns, 2009). In recent years, measured values, such as synchronization likelihood (Stam and Van Dijk, 2002) and the phase lag index (PLI) (Stam et al., 2007), have been used as an evaluation method for phase synchronization to solve the problem of volume conduction as a cause for the detection of spurious synchronizations (Nunez et al., 1997; Nolte et al., 2004). By utilizing this advantage of the PLI within EEG, alterations in functional connectivity under pathological conditions have been revealed in previous studies (Engels et al., 2015; Ueda et al., 2020; Nobukawa et al., 2020a). For example, children with ADHD were reported to have a lower gamma PLI than children with typical development (Ueda et al., 2020); AD is associated with a reduced alpha, beta, and gamma PLI compared with that observed in healthy controls (Nobukawa et al., 2020a). Likewise, patients with schizophrenia reportedly demonstrate a reduced PLI of the beta band in the frontal region and a reduced PLI of the gamma band throughout the scalp (Takahashi et al., 2018). The PLI has also been used to assess frequency dependence in children with ASD (Takahashi et al., 2017). Furthermore, the PLI can capture functional connectivity within high cognitive functions among healthy older participants (Nobukawa et al., 2020b). PLI is robust against artifacts such as body and eye movements, and muscle activation thus the influence of artifacts on PLI is relatively small, because the major parts of this influence lie in the amplitude space of signals, while PLI estimates phase-based functional connectivity (Stam et al., 2007). However, in the higher frequency gamma band range, artifacts due to muscle activity are larger compared to slower frequency ranges (Whitham et al., 2007, 2008); therefore, there may be issues with PLI estimation accuracy in the gamma range (Lau et al., 2012; Engels et al., 2015).

In recent trends within neural activity analysis, multiple spatio-temporal profiles of neural activity (which combine profiles obtained by several evaluation methods) are integrated via machine learning; subsequently, analyses detecting the pathology of several psychiatric disorders and estimating the ability of brain function have been conducted with higher accuracy as compared with using a single profile (reviewed in Vu et al., 2018). In particular, informative studies have been conducted combining profiles of functional connectivity and temporal complexity (Ghanbari et al., 2015; Nobukawa et al., 2020a). Studies have also reported a complementary relationship between functional connectivity and neural complexity (Ghanbari et al., 2015; Nobukawa et al., 2020a). In patients with ASD, increasing (or decreasing) complexity decreases (or increases) functional connectivity, suggesting that the functional connectivity and complexity are complementary (Ghanbari et al., 2015). For patients with AD, the relationship between functional connectivity and complexity shows different temporal scales and region-specific dependencies in both healthy participants and among patients with AD, suggesting that the relationship between functional connectivity and complexity may reflect the complex pathological process occurring within AD (Nobukawa et al., 2020a). However, to the best of our knowledge, an approach combining functional connectivity and the complexity of neural activity has not been evaluated under healthy conditions. Even in the healthy aging process, brain functions, such as attention, memory, and cognitive functions, degrade over time (Birren and Fisher, 1995). Therefore, in a super-aging society, the alteration of spatial-temporal neural activity owing to aging is considered crucial for interventions for the prevention of brain dysfunction.

Thus, we hypothesized that the complementary relationship between functional connectivity and complexity could improve the ability to detect alteration of spatiotemporal patterns within EEG with respect to the aging process. In this study, MF and MSE analyses were performed to evaluate the temporal complexity profiles, and PLI analyses evaluating the unique profile of functional connectivity were performed based on EEG among younger and older participants. Subsequently, these profiles were combined via machine learning methodology.

## 2. Materials and Methods

### 2.1. Participants

A total of 32 healthy younger people (15 males, 17 females; average age, 23.9 years; standard deviation [SD], 4.7 years; age range, 20–35 years) and 18 healthy older people (7 males, 11 females; average age, 57.5 years; SD, 4.7 years; age range, 51–67 years) were enrolled in this study. These groups were sex-matched (χ^2^ = 0.30, *p* = 0.59). The older participants were all non-smokers and were not on any medications. Participants with medical or neurological conditions (including epilepsy or head trauma occurring in the past), as well as those with a history of alcohol or drug dependence, were excluded from the current study. All the participants provided their written informed consent following an explanation of study procedures as well as risks and benefits by study personnel. This study was approved by the Ethics Committee of Kanazawa University and was conducted in accordance with the principles of the Declaration of Helsinki and its later amendments. The EEG data used in this study evaluated the dynamics of phase synchronization (Nobukawa et al., 2019a).

### 2.2. EEG Recordings

Methods for recording and pre-processing EEG data have been reported and established in previous research (Mizuno et al., 2010). Specifically, the participants in the current study sat in a soundproof recording room, and their EEG was measured under controlled room lighting conditions. For EEG measurement, 16 electrodes (Fp1, Fp2, F3, F4, C3, C4, P3, P4, O1, O2, F7, F8, Fz, Pz, T5, and T6) were used; this system was based on the recommended electrode arrangement under the international 10–20 system. EEG activity was measured with reference to the binaural connection. The EEG-4518 monitor used for electroencephalogram measurements in this study was manufactured by Nihon Kohden Co., Ltd. (Tokyo, Japan). The sampling frequency was 200 Hz for the recording. The electrode/skin conductance impedance was controlled to within less than 5kΩ for each electrode. Participants' electroencephalogram signals were measured for 10–15 min in a resting state with the eyes closed. Researchers visually inspected the participants' arousal using a video surveillance system; participants were asked to close their eyes, and researchers confirmed that only awake epochs were measured. If the alpha and theta oscillations became weaker or stronger, compared with ones at beginning stage of the recording, this duration was not used for evaluation, because this duration belonged to the light sleep stage. Additionally, the EEG signals were visually assessed to identify artifacts, such as muscle activity, blinks, and eye movement; consequently, 60-s (12,000 data points) of artifact-free time-series segments within the EEG signals recorded in the awake state with eyes closed were identified. For each epoch, bandpass filtering with the range of 2.0–60 Hz was applied. The first and last 5-s period (1,000 data point) in each bandpass-filtered epoch were removed to avoid transient behaviors produced by the bandpass filtering process. MSE and MF analyses were performed for 50 consecutive seconds (i.e., 10,000 data points) of epochs. In the PLI analyses, values decrease with increasing epoch length (Fraschini et al., 2016); therefore, it is difficult to identify changes with an increasing epoch length. In addition, using short epoch lengths makes it impossible to capture information on slow frequency components. To balance these considerations, the PLI analysis divided 50 consecutive seconds (10,000 data points) into 10 epochs of 5 s each (Takahashi et al., 2017, 2018; Nobukawa et al., 2020a,b).

### 2.3. Multi-Fractal Analysis

**Figure 1:**
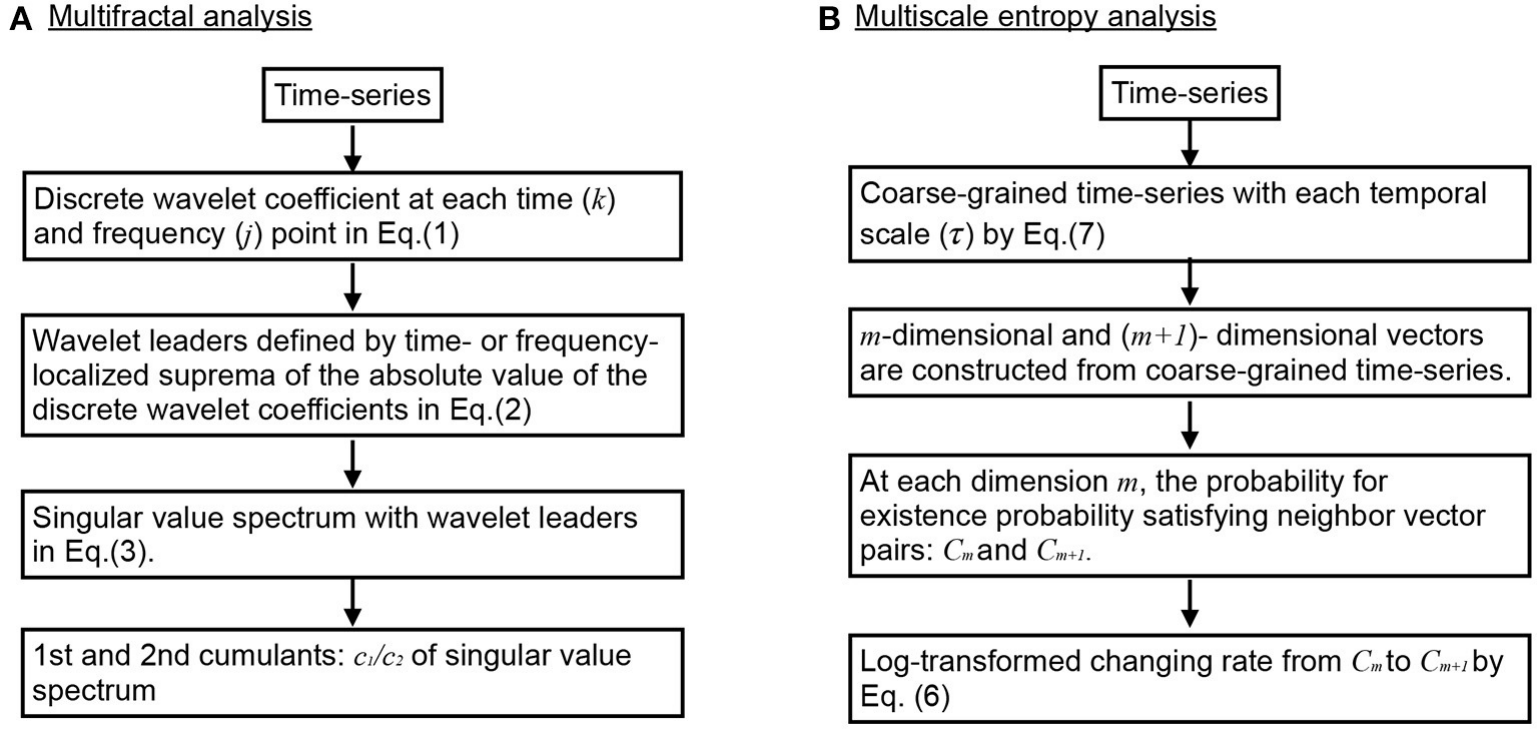
Overview of flow for complexity analysis. **(A)** Multifractal (MF) analysis. **(B)** Multiscale entropy (MSE) analysis.

The overview of flow for multi-fractal analysis is shown in Figure 1A. In MF analyses, wavelet leaders derived from the coefficients of the discrete wavelet transform are widely used (Jaffard et al., 2006; Wendt and Abry, 2007). MF analysis is an analysis method that uses the Hölder index to represent the fractal dimension of the partial structure that characterizes the structure of data *X* via spectrum data. The discrete wavelet coefficient of the discrete signal *X*(*t*) is given by Equation (1).


(1) $$\begin{array}{l} d_{X}\left(j,k\right)=\int_{R}X\left(t\right)2^{j},\psi_{0}\left(2^{-j}t-k\right)dt\;\left(j=1,2,...,k=1,2,...\right) \end{array}$$

Here, ψ_0_ is a compact-supported mother wavelet function. The Equation (2) shows one-dimensional wavelet leaders which are time- or frequency-localized suprema of the absolute value of the discrete wavelet coefficients *d*_*X*_(*j, k*):


(2) $$\begin{array}{l} L_{x}\left(j,k\right)=\underset{\lambda^{\prime }\subset 3\lambda_{j,k}}{\text{sup}}|d_{X}\left(\lambda^{\prime }\right)| \end{array}$$

Here, $\lambda =\lambda_{j,k}=\left[k2^{-j},\left(k+1\right)2^{-j}\right]$ represents the time interval of the scale 2^−*j*^. Additionally, 3λ_*j,k*−1_ = ∪λ_*j,k*_ ∪ λ_*j, k*+1_ represents the adjacent time (Wendt and Abry, 2007). The spectrum of singularity of *L*_*X*_ is defined by Equation (3) with wavelet leaders (Jaffard et al., 2006; Wendt and Abry, 2007).


(3) $$\begin{array}{l} D\left(h\right)=\underset{q\neq 0}{\text{inf}}\left(1+qh-\zeta_{L}\left(q\right)\right) \end{array}$$

Here, *h* indicates the Hölder index. Also, *q* indicates the moment of the wavelet leaders. The scaling index ζ_*L*_(*q*) is defined by Equation (4). The wavelet leader structure function *S*_*L*_(*q, j*) is defined by Equation (5).


(4) $$\begin{array}{l} \zeta_{L}\left(q\right)=\underset{j\to \infty }{\text{lim inf}}\left(\frac{{\text{log}}_{2}S_{L}\left(q,j\right)}{\log_{2}2^{-j}}\right) \end{array}$$

(5) $$\begin{array}{l} S_{L}\left(q,j\right)=\frac{1}{n_{j}}\overset{n_{j}}{\underset{k=1}{\sum }}|L_{X}\left(j,k\right){|}^{q} \end{array}$$

Here, *n*_*j*_ indicates the number of samples of *X* when the scale is 2^*j*^. As the Hölder index *h* approaches 1.0, the time-series shape becomes more differentiable. However, as the Hölder index *h* approaches 0, the time-series shape becomes nearly discontinuous. A signal is monofractal if the scaling index ζ_*L*_(*q*) is a linear function and *D*(*h*) converges to a particular *h*. Contrastingly, the fact that the signal is multi-fractal indicates a scaling index, where ζ_*L*_(*q*) deviates from linearity and *D*(*h*) is widely distributed in *h*. In this study, to capture the profile of *D*(*h*), the primary cumulant *c*_1_ of *D*(*h*) was used as an indicator of the smoothness of the entire time series signal, and the secondary cumulant *c*_2_ was used as an index evaluating the local fluctuation of the time-series signal. For the multi-fractal time series, *D*(*h*) is distributed around *c*_1_. Therefore, the degree of distribution of *D*(*h*) reflects the multi-fractal property, which corresponds to |*c*_2_|. The time-series with large (small) multi-fractality (|*c*_2_|) exhibits intermittent and transient behavior with large (small) amplitude (Ihlen, 2012); while, the complexity notified by *c*_1_ reflects the degree of complexity for temporal behavior in entire time-range, instead of intermittent behavior (see Supplementary Material). In this study, multi-fractal analysis was performed using the wavelet toolbox in MATLAB (https://jp.mathworks.com/products/wavelet.html; MathWorks, Natick, MA, USA).

### 2.4. Multi-Scale Entropy Analysis

The overview of flow for MSE analysis was shown in Figure 1B. MSE analysis was used to assess the temporal scale dependence for EEG time series complexity (Costa et al., 2002). The time-series sample entropy of the random *Z*-score variables {*x*_1_, *x*_2_, ..., *x*_*N*_} is given by Equation (6).


(6) $$\begin{array}{l} h\left(r,m\right)=-\log\frac{C_{m+1}\left(r\right)}{C_{m}\left(r\right)}. \end{array}$$

Here, *C*_*m*_(*r*) is the probability of $|{\text{x}}_{i}^{m}-{\text{x}}_{j}^{m}|<r\left(i\neq j,i,j=1,2,...\right)$ among all pairs of *i* and *j*. ${\text{x}}_{i}^{m}$ indicates an *m*-dimensional vector ${\text{x}}_{i}^{m}=\left\{x_{i},x_{i+1},...,x_{i+m-1}\right\}$. In the MSE analysis, {*x*_1_, *x*_2_, ..., *x*_*N*_} is calculated using Equation (7) for coarse-grained time series *y*_*j*_.


(7) $$\begin{array}{l} x_{j}=\frac{1}{\tau }\overset{j_{\tau }}{\underset{i=\left(j-1\right)\tau +1}{\sum }}y_{i}\left(1\leq j\leq \frac{N}{\tau }\right). \end{array}$$

Here, {*y*_1_, *y*_2_, ..., *y*_*N*_} represents the observed signals. τ(τ = 1, 2, ...) represents the temporal scale. In this study, we set *m* = 2 and *r* = 0.2 (Costa et al., 2002) and MSE analysis was performed using the Physio Toolkit toolbox in MATLAB (http://physionet.incor.usp.br/physiotools/sampen/).

### 2.5. Phase Lag Index Analysis

The PLI was obtained to measure phase synchronization, and the characteristics of the synchronization signal were quantitatively estimated. The EEG signal was divided into five frequency bands: the delta (2–4 Hz), theta (4–8 Hz), alpha (8–13 Hz), beta (13–30 Hz), and gamma bands (30–60 Hz). Here, several studies showed that artifacts in the gamma band due to muscle activity is larger compared with slower frequency bands (Whitham et al., 2007, 2008). Therefore, through visual examination of EEG signals, the time including muscle activity was avoided in the evaluation epochs. Each band division divides the signal at time *t*, and the point ϕ_*a*_ is indicated by phase ϕ_*a*_(*t*) and amplitude *Aa*(*t*) using the Hilbert transform. In addition, the phase difference Δϕ_*ab*_(*t*_*i*_) observed between signals with two different points *a* and *b* at time *t*_*i*_ is given by Equations (8) and (9) (Stam et al., 2007).


(8) $$\begin{array}{l} \Delta \phi_{ab}\left(t_{i}\right)=\phi_{a}\left(t_{i}\right)-\phi_{b}\left(t_{i}\right) \end{array}$$

(9) $$\begin{array}{l} \Delta \phi_{\text{mod}}\left(t_{i}\right)=\Delta \phi_{ab}\left(t_{i}\right)\text{mod}2\pi \end{array}$$

The PLI of the signal between the two points *a* and *b* for the duration *T* is given by Equation (10).


(10) $$\begin{array}{l} PLI_{ab}=\left|\frac{1}{T}\overset{T}{\underset{i=0}{\sum }}\text{sign}\left(\Delta \phi_{\text{mod}}\left(t_{i}\right)\right)\right| \end{array}$$

When signals with the same source are observed at different points, Δϕ_*ab*_(*t*_*i*_) is 0 and Δϕ_mod_(*t*_*i*_) = 0; subsequently the *PLI*_*ab*_ value becomes 0. In addition, the observation at the point on the opposite side of the electric dipole is defined as Δϕ_*ab*_(*t*_*i*_) = π within Equation (8) in cases where the signal source is assumed to follow the dipole model. This yields *PLI*_*ab*_ = 0. The average PLI of any electrode *a* via another electrode *b* = 1, 2, ..., *K*(*b* ≠ *a*) (called the node strength; NS) is given by Equation (11). Here, *K* represents the total number of electrodes *K* = 16.


(11) $$\begin{array}{l} NS_{a}=\frac{1}{K-1}\overset{K}{\underset{b=1,b\neq a}{\sum }}PLI_{ab} \end{array}$$

### 2.6. Statistical Analysis

For *c*_1_ and |*c*_2_|, repeated-measures analysis of variance (ANOVA) was performed to determine statistically significant differences between the younger and older groups. Age group was used as an inter-subject factor, and the 16 electrodes from Fp1 to T6 were used as intra-subject factors. The ANOVA results were represented by *F* values based on intra-group and inter-group variance comparisons. Greenhouse-Geisser adjustments were applied to the degrees of freedom. The α = 0.05 bilateral level was used; this was considered a statistically significant criterion for avoiding type I errors. A *post-hoc* *t*-test was subsequently used to evaluate the main effect between the younger and older age groups and effect of the interactions per electrode. Here, Benjamini-Hochberg false discovery rate (FDR) correction was applied to the *t* value for multiple comparisons of *c*_1_ and |*c*_2_| (*q* < 0.05) (16 *p* values: 16 electrodes).

In the ANOVA for sample entropy, age group was used as an inter-subject factor, and the 16 electrodes from Fp1 to T6 and a temporal scale were used as intra-subject factors. A *post-hoc* *t*-test was subsequently used to evaluate the main effect between the younger and older groups and effects of interaction for the electrodes and temporal scales. The α = 0.05 bilateral level was used. FDR correction was applied to the *t* scores for multiple comparisons (*q* < 0.05) (480 *p* values: 16 electrode × 30 scales).

In ANOVA for NS at each frequency band, age group was used as an inter-subject factor, and the 16 electrodes (from Fp1 to T6) were used as intra-subject factors. A *post-hoc* *t*-test was subsequently used to evaluate the main effect between the younger and older groups and effect of the interaction for the electrodes. The α = 0.05 bilateral level was used. FDR correction was applied to the *t* scores for multiple comparisons (*q* < 0.05) (80 *p* values: 16 electrodes × 5 frequency bands). The *t*-test was used for electrode-pair-wise group comparison of PLI between the younger and older groups. With a control for multiple comparisons, FDR correction was applied to the *t* scores (*q* < 0.05) (600 *p* values: 120 electrode pairs × 5 bands).

Older participants were classified using the receiver operating characteristics (ROC) curve. A logistic regression model based on sample entropy, *c*_1_, |*c*_2_|, and the NS of the PLI was used to identify older participants. Here, the logistic regression model outputs the “older participants” discrimination probability for each participant. The true/false positive rate at each threshold of discrimination probability from 0 to 1.0 for both groups was then measured. Principal component analysis was used as a pre-treatment for dimensionality reduction, and logistic regression based on *c*_1_, |*c*_2_|, sample entropy, and the NS of the PLI was implemented. The accuracy of discrimination was evaluated using the area under the ROC curve (AUC). We also used 5-fold cross-validation to prevent overfitting; AUC = 1.0 corresponds to perfect discrimination, and AUC = 0.5 corresponds to random discrimination. Here, the principal component analysis was conducted within cross validation (Shim et al., 2021) to avoid the inaccurate estimation of performance of discrimination. AUC values were averaged among 20 trials to choose tested and evaluated data set in 5-fold cross-validation and their standard deviations (SD) were also derived.

To evaluate the relationship between NS and complexity, we used Spearman's correlation *R* between NS and the complexity indexes (*c*_1_, |*c*_2_|). To control the multiple comparison, FDR correction was applied these *R*-scores (*q* < 0.05) (16 *p* values: 16 electrodes).

## 3. Results

### 3.1. Multi-Fractal Analysis

**Figure 2:**
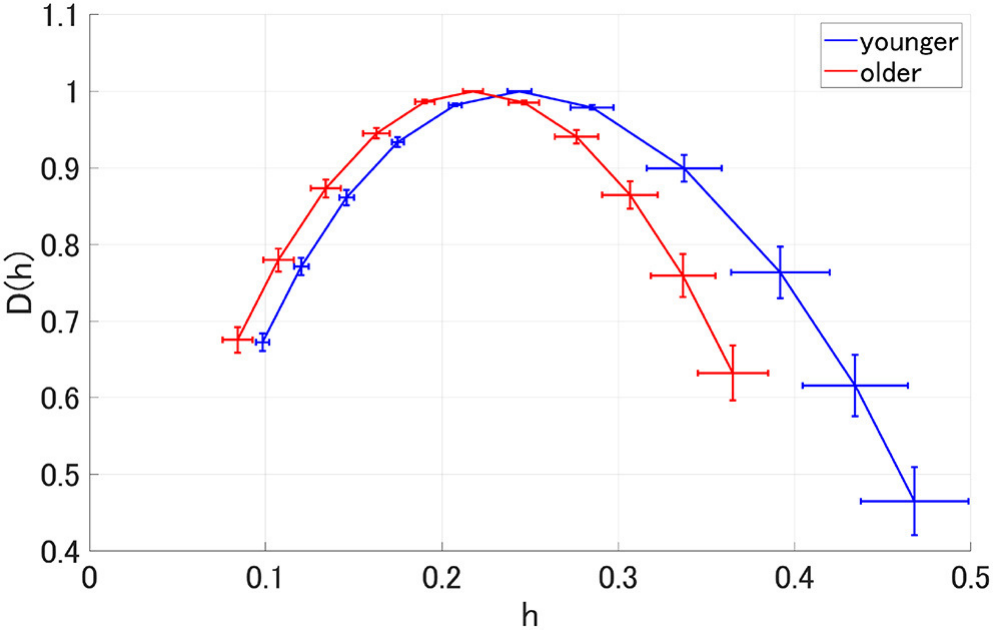
Spectrum of singularity for the older and younger groups. This figure shows the mean and standard deviation of each group for *D*(*h*) and *h*, respectively. Owing to the wide distribution observed here, these results are thought to reflect the multifractal characteristics of the EEG signals for both groups.

**Figure 3:**
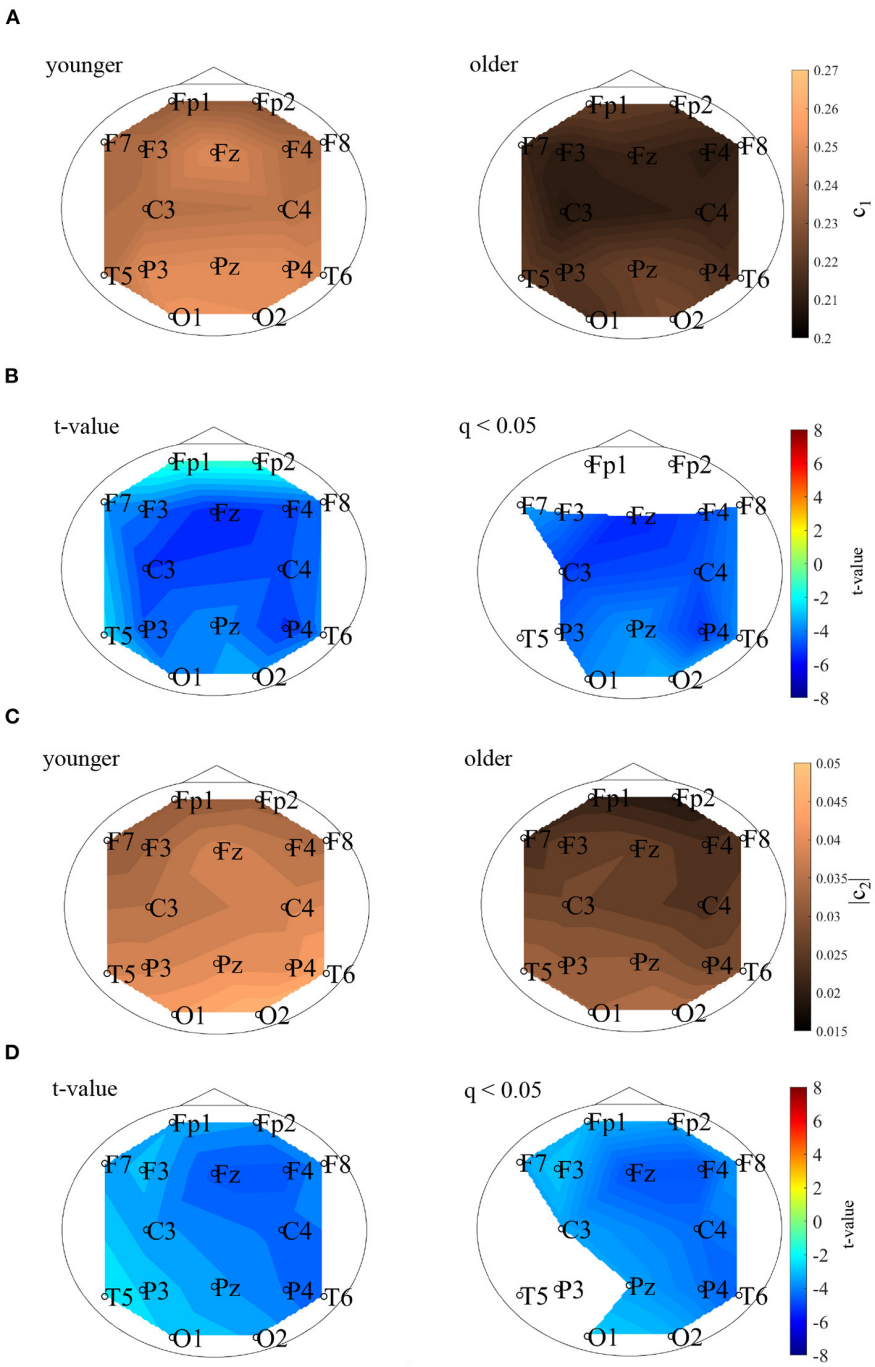
**(A)** 1st cumulant of the spectrum of singularity *c*_1_. The mean values of *c*_1_ in the younger (left) and older (right) groups. **(B)** *t* values comparing the younger and older groups. Warm (cold) colors represent higher (smaller) *c*_1_ values for older versus younger participants. The left and right of the figure correspond to the *t*- and *t*-values satisfying the false discovery rate (FDR) correction criteria *q* < 0.050. The *c*_1_ value for the older group, which had statistically significantly lower values at F3, Fz, F4, F7, F8, C3, C4, P3, Pz, P4, T6, O1, and O2, and is shown here. **(C)** Absolute value of 2nd cumulant of the spectrum of singularity |*c*_2_|. The mean values of |*c*_2_| in the younger (left) and older (right) groups are shown here. **(D)** *t*-values comparing the older and younger groups. Warm (cold) colors represent higher (smaller) |*c*_2_| values for older versus younger participants. The left and right correspond to the *t*- and *t*-values satisfying the FDR correction criteria *q* < 0.050. The |*c*_2_| of the older group had statistically significantly lower values at Fp1, Fp2, F3, Fz, F4, F7, F8, C3, C4, Pz, P4, T6, O1, and O2.

MF analysis was performed in the younger and older participants. Figure 2 shows the mean and standard deviation for each group with respect to *D*(*h*) and *h*. Owing to its wide distribution, this analysis is thought to reflect the multi-fractal characteristics (Sikdar et al., 2018) of the EEG signals for both groups. Table 1 shows the repeated-measures ANOVA results of the first (*c*_1_) and second (|*c*_2_|) cumulants within a singular spectrum. A strong main effects were observed for *c*_1_ and |*c*_2_|. The mean values of *c*_1_ and |*c*_2_| in the older and younger groups, as well as the results of a *post-hoc* *t*-test between the older and younger groups, are shown in Figure 3. The *post-hoc* *t*-test revealed that the value of *c*_1_ was statistically significantly lower for the older participants at 13 electrodes (F3, Fz, F4, F7, F8, C3, C4, P3, Pz, P4, T6, O1, and O2) (*q* < 0.050). In addition, the results showed that the value of |*c*_2_| was statistically significantly lower for the older participants at 14 electrodes (Fp1, Fp2, F3, Fz, F4, F7, F8, C3, C4, Pz, P4, T6, O1, and O2) (*q* < 0.050). The results of the MF analysis demonstrated that aging increases complexity (shown by less smoothness) and decreases multi-fractality. The time-series with large (small) multi-fractality exhibits intermittent and transient behavior with large (small) amplitude (Ihlen, 2012). Meanwhile, complexity reflects the degree of complexity for temporal behavior in entire the time-range, rather than intermittent behavior. Therefore, EEG signal in older subjects corresponds to homogeneous and highly complex temporal behaviors.

**Table 1:** Younger vs. older repeated measure ANOVA analysis results [*F* value (*p* value, partial η^2^)] in multifractal (MF) analysis.

	**Group**	**Group × nodes**	**Degree of freedom (ϵ)**
***c*_1_**	***F* = 25.25 (*p* < 0.001, η^2^ = 0.345)**	*F* = 1.73 (*p* = 0.13, η^2^= 0.035)	5.06 (ϵ = 0.034)
**|*c*_2_|**	***F* = 22.23 (*p* < 0.001, η^2^ = 0.317)**	*F* = 1.73 (*p* = 0.11, η^2^ = 0.035)	6.04 (ϵ = 0.035)

*F and p values with p < 0.05 are represented by bold characters. Degree of freedom and Greenhouse-Geisser adjustments ϵ in the interaction for group × nodes are also shown*.

### 3.2. Multi-Scale Entropy Analysis

**Figure 4:**
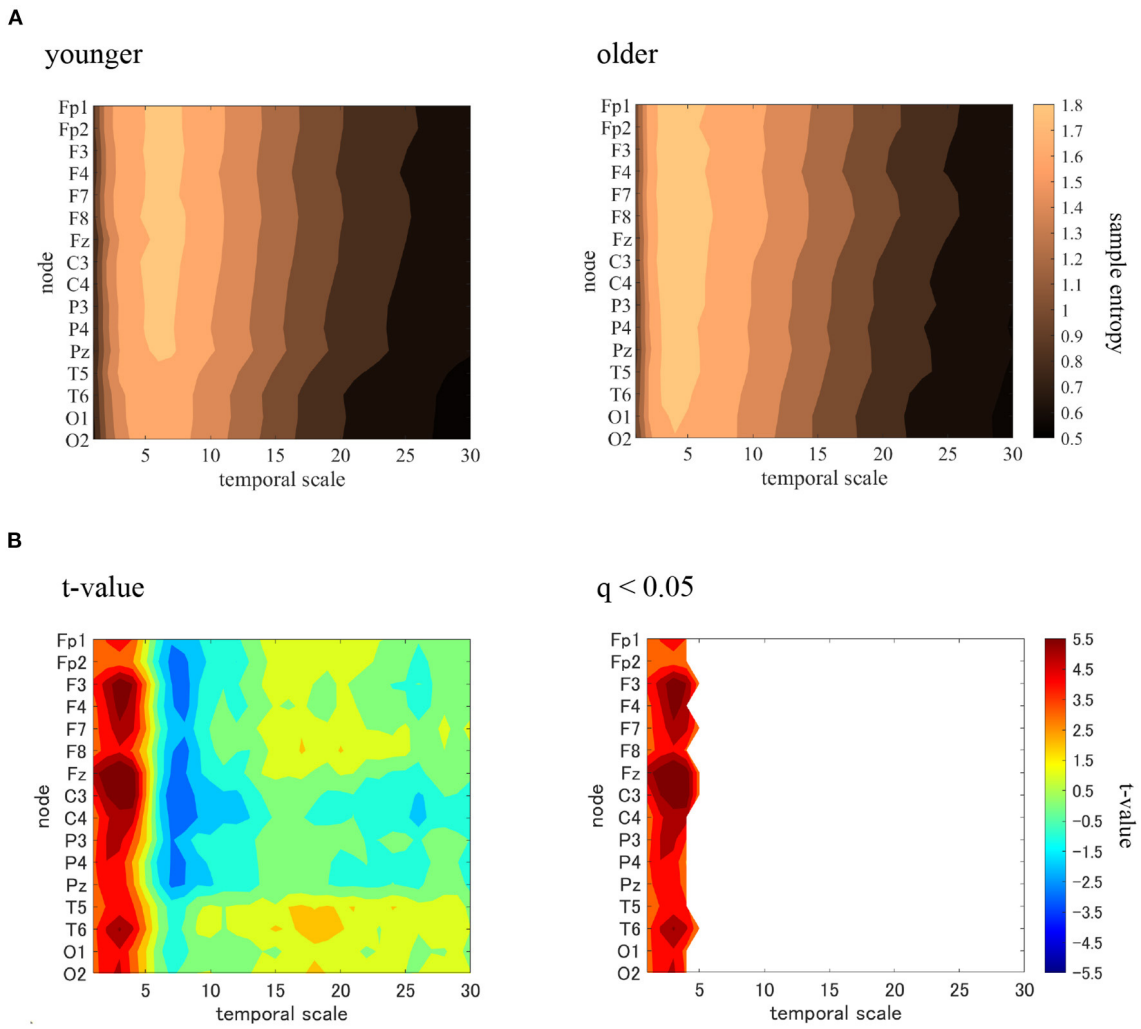
Multi-scale entropy analysis in younger and older groups. The horizontal axis represents the temporal-scale factor, τ. **(A)** Mean values of sample entropy from 1 (0.005 s) to 30 (0.15 s) scale factors in younger (left part) and older (right part) participants are shown here. **(B)** *t*-values comparing the older and younger groups are shown here as well (left part). Warm (cold) colors represents a higher (smaller) sample entropy value for older individuals than for younger participants. The observed *t*-value satisfies the FDR correction criterion *q* < 0.050 (right part). A statistically significantly higher sample entropy of the low temporal-scale regions 1 to 5 (0.005–0.025 s) is depicted here.

MSE analysis was performed in the younger and older participants. Table 2 shows the repeated-measures ANOVA results for the MSE analysis. We found that no main effect was observed, although there were interactions in the group × scale and the group × node × scale. The mean values of sample entropy in the older and younger groups, as well as the results of a *post-hoc* *t*-test between the older and younger groups, are shown in Figure 4. The results demonstrated a statistically significantly higher sample entropy for the older participants (*q* < 0.050) in the temporal-scale region of 1 to 5 (0.005 to 0.025 s at all electrodes). The results of the MSE analysis demonstrated that aging increases complexity on a fast temporal scale.

**Table 2:** Younger vs. older repeated–measures ANOVA results [*F* value (*p* value, partial η^2^)], degree of freedom and Greenhouse-Geisser adjustments ϵ in multi scale entropy (MSE) analysis.

**Group**	**Group × node**	**Group × scale**	**Group × node × scale**
*F* = 3.37 (*p* = 0.073, η^2^ = 0.066)	*F* = 1.51 (*p* = 0.21, η^2^ = 0.030, degree of freedom: 3.62, ϵ = 0.242)	***F* = 19.93 (*p* < 0.001, η^2^ = 0.293 degree of freedom: 2.94, ϵ = 0.102)**	***F* = 1.949 (*p* = 0.020, η^2^ = 0.039 degree of freedom: 13.898, ϵ = 0.032)**

*F and p values with p < 0.05 are represented by bold characters*.

### 3.3. Phase Lag Index Analysis

**Figure 5:**
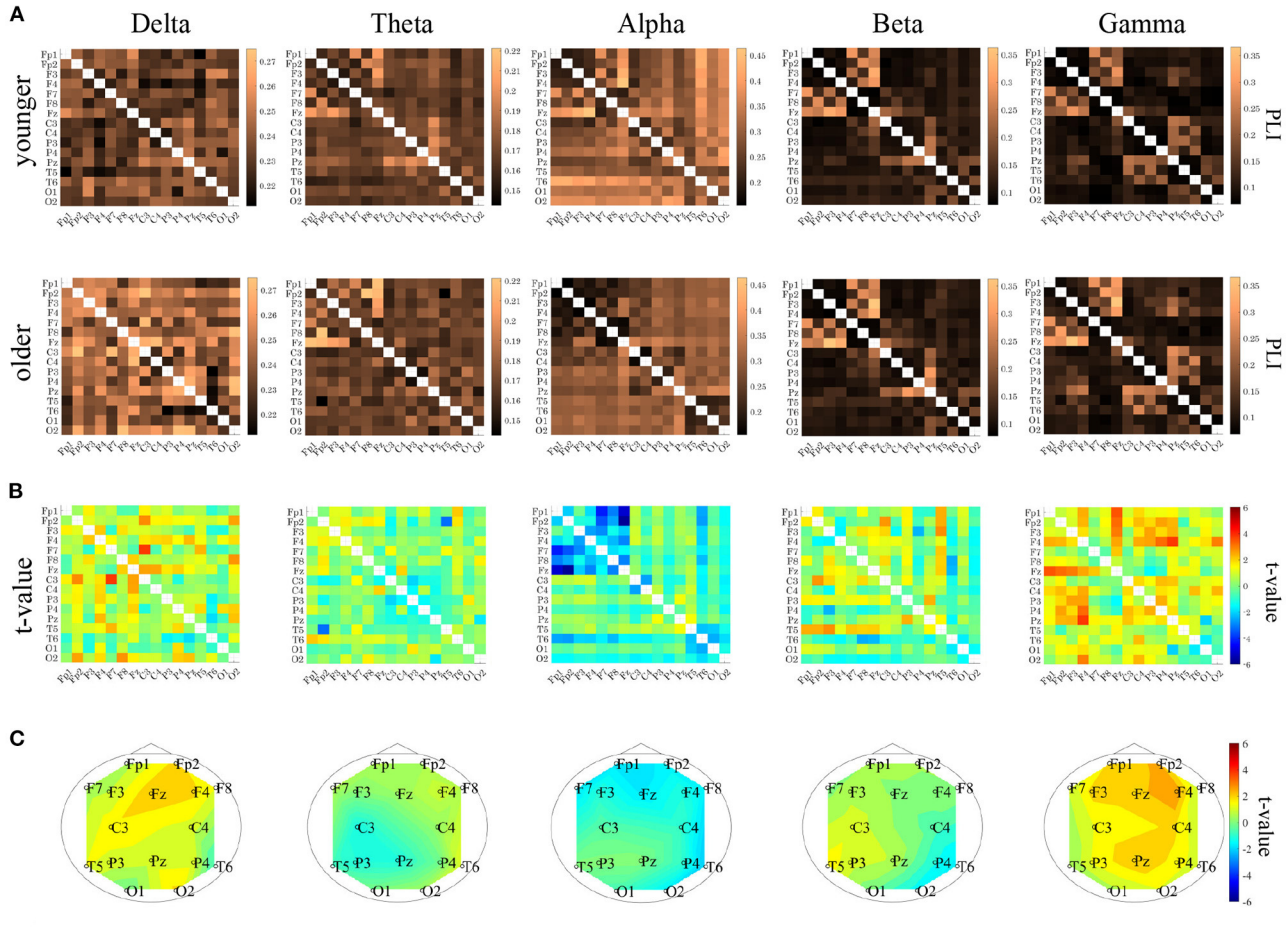
**(A)** Mean values of phase lag index (PLI) for each band in younger (upper parts) and older (lower parts) groups. **(B)** *t*-values between the younger and older groups (upper parts). The warm (cold) colors represents higher (smaller) PLI values in the older group than in the younger group. (lower parts). **(C)** *t*-values for the node strength (NS) of PLI between the older and younger groups. Warm (cold) colors represent a higher (smaller) NS for older versus younger individuals. Although no statistically significant differences satisfying with FDR criteria *q* < 0.05 were observed between the older and younger groups, relatively higher NS at delta and gamma band in the older group was observed. In PLI among pair-wise electrodes, no statistically significant differences satisfying with FDR criteria (*q* < 0.05) were observed between the older and younger groups.

PLI analysis was performed on younger and older participants. Table 3 shows the ANOVA analysis results for the NS of the PLI for each band among younger and older participants. The results indicated that there was a main effect in the delta and gamma bands and that there was an interaction with respect to the group × node in the alpha, beta, and gamma bands. The *post-hoc* *t*-test results for the NS are shown in Figure 5C. Although no statistically significant differences satisfying with FDR criteria *q* < 0.05 were observed between the older and younger groups, relatively higher NS at delta and gamma band in the older group was observed. Regarding the PLI among pair-wise electrodes, the mean values of the PLI in the older and younger groups, as well as the results of *t*-tests between the older and younger groups, are shown in Figures 5A,B. No statistically significant differences satisfying with FDR criteria (*q* < 0.05) were observed between the older and younger groups.

**Table 3:** Younger vs. older repeated-measure ANOVA analysis results [*F* value (*p* value, partial η^2^)] in the node strength (NS) of phase lag index (PLI).

	**Group**	**Group × nodes**	**Degree of freedom (ϵ)**
delta	***F* = 4.18 (*p* = 0.046, η^2^ = 0.80)**	*F* = 1.26 (*p* = 0.262, η^2^ = 0.026)	8.183 (ϵ = 0.546)
theta	*F* = 0.04 (*p* = 0.833, η^2^ = 0.001)	*F* = 0.98 (*p* = 0.453, η^2^ = 0.020)	8.948 (ϵ = 0.597)
alpha	*F* = 1.09 (*p* = 0.301, η^2^ = 0.022)	***F* = 3.95 (*p* < 0.001, η^2^ = 0.076)**	5.899 (ϵ = 0.393)
beta	*F* = 0.097 (*p* = 0.757, η^2^ = 0.002)	***F* = 2.89 (*p* = 0.006, η^2^ = 0.057)**	7.060 (ϵ = 0.471)
gamma	***F* = 4.17 (*p* = 0.047, η^2^ = 0.080)**	***F* = 3.35 (*p* = 0.002, η^2^ = 0.065)**	6.785 (ϵ = 0.452)

*F and p values with p < 0.05 are represented by bold characters. Degree of freedom and Greenhouse-Geisser adjustments ϵ in the interaction for group × nodes are also shown*.

### 3.4. Correlation Analysis Between Complexity and Functional Connectivity

**Figure 6:**
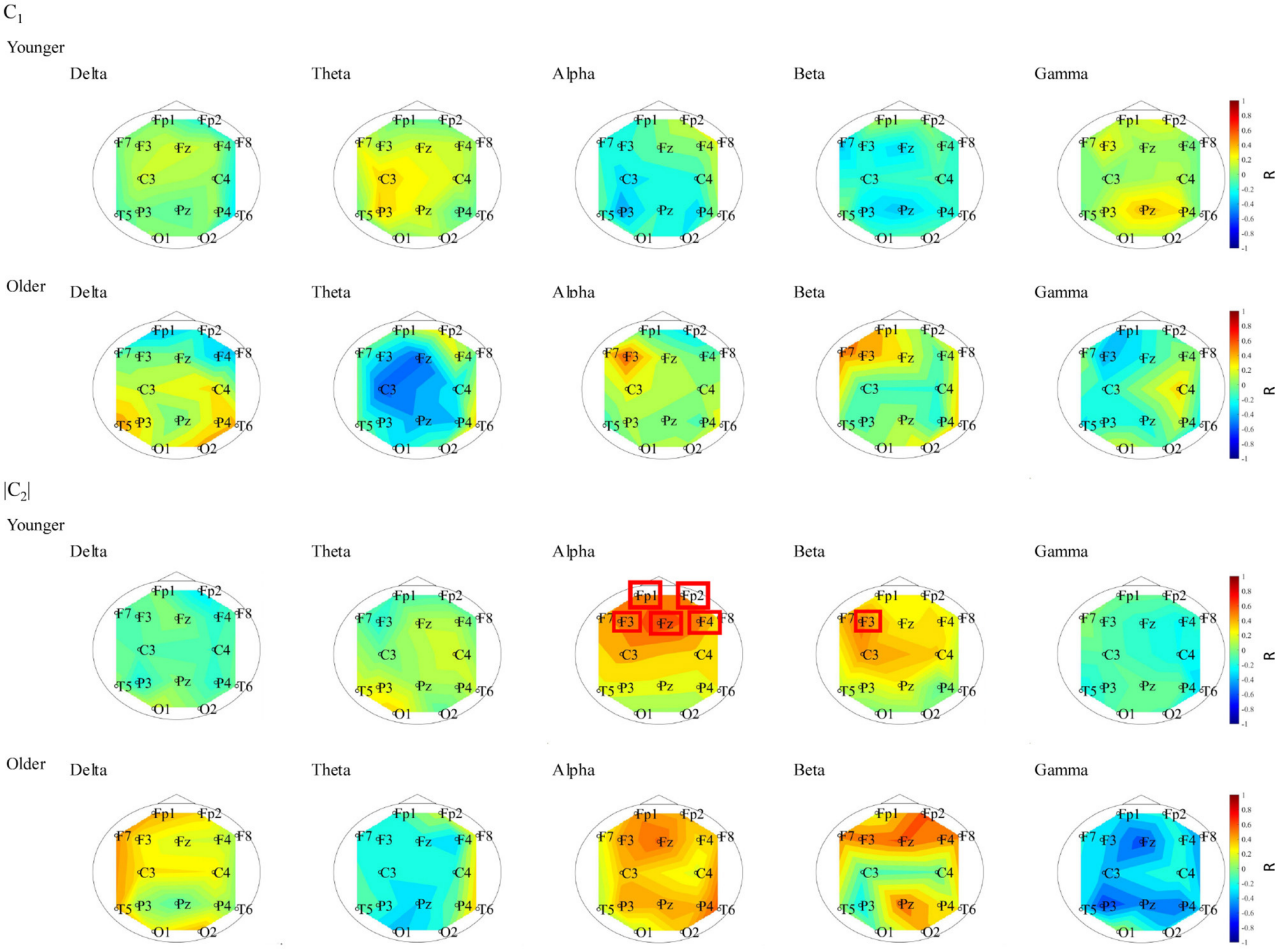
Spearman's rank correlation coefficient *R* between NS of PLI and *c*_1_ in younger (1st line) and older (2nd line) groups. The correlation values did not meet the FDR correction criteria of *q* < 0.050. Spearman's rank correlation coefficient *R* between NS of PLI and |*c*_2_| in younger (3rd line) and older (4th line) groups. The electrodes with correlation values to meet the FDR correction criteria of *q* < 0.050 are surround with a line. In younger group, positive correlation with NS at alpha and |*c*_2_| at Fp1, Fp2, F3, F4, and Fz and positive correlation with NS at beta band and |*c*_2_| at F3 were confirmed.

**Figure 7:**
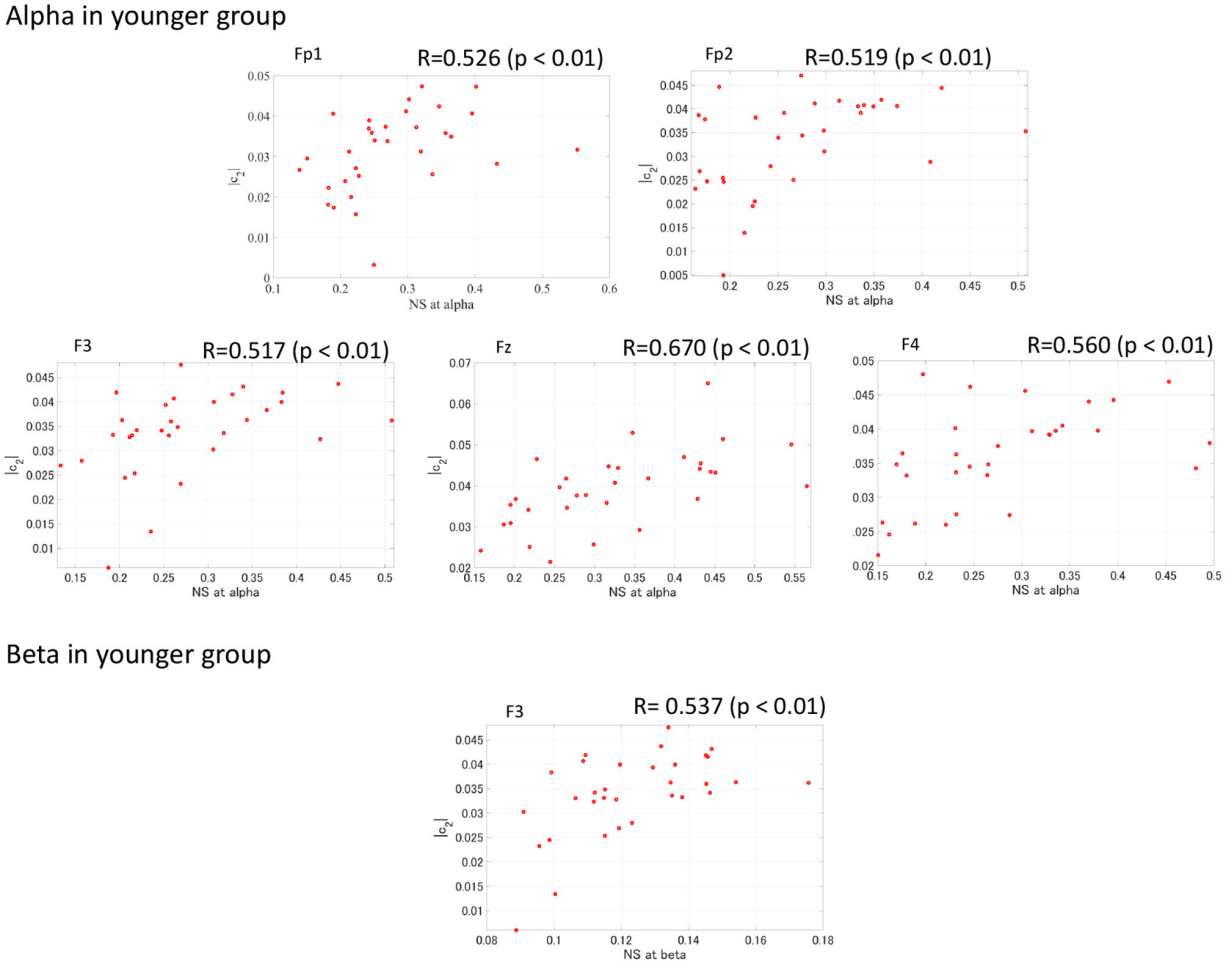
Scatter plot between NS of PLI at alpha/beta bands and |*c*_2_| in younger group where Spearman's rank correlation *R* coefficient satisfies with FDR correction criteria of *q* < 0.050 in Figure 6. The significant large positive correlations were observed.

To evaluate the relationship between complexity and functional connectivity, a correlation analysis was performed, using Spearman's correlation, between NS and the complexity indexes (*c*_1_, |*c*_2_|). Figure 6 shows the Spearman's correlations between NS of PLI and *c*_1_ and between NS of PLI and |*c*_2_| in both the younger and older groups. The correlation with *c*_1_ did not meet the FDR correction criteria of *q* < 0.050; while there were positive correlations with NS at alpha and |*c*_2_| at Fp1, Fp2, F3, F4, and Fz as well as NS at beta band and |*c*_2_| at F3 in younger group. In Figure 7, the scatter plots at these electrodes were shown, significantly large positive correlations were observed. Therefore, large node strength might lead the intermittent and transient behavior reflecting |*c*_2_| in local neural activity, instead of steady neural variability reflecting *c*_1_.

### 3.5. ROC Curve Analysis

To evaluate the classification ability for *c*_1_, |*c*_2_|, and the PLI, we evaluated the ROC. We observed a statistically significantly large sample entropy in the older group for time scales of 1 to 5, as shown in Figure 4. Therefore, the sample entropy was averaged in this temporal region for the purpose of clarification. Table 4 shows the results of the ROC in cases with the first-third principal components of *c*_1_ and |*c*_2_|, as well as sample entropy. In the results shown in Table 4, *c*_1_ had the highest value (AUC = 0.86). Table 5 shows the results of the ROC in cases with the first-third principal components for the NS of the PLI in the delta, theta, alpha, beta, and gamma bands. In the results shown in Table 5, NS at alpha had the highest value (AUC = 0.84). Table 6 shows the results of the ROC in cases with the first-third principal components of the combination of *c*_1_ and |*c*_2_| and the combination of the NS at alpha, as well as *c*_1_, |*c*_2_| and sample entropy. Almost AUC values increased by combining the NS at alpha, *c*_1_, |*c*_2_|, and sample entropy in comparison with cases using a single index, as shown in Tables 4, 5. While, at the other bands, AUCs in the NS are significantly lower in comparison with the complexity indexes (*c*_1_, *c*_2_, and sample entropy) (see Tables 4, 5). Therefore, AUCs in the case with combinations of NS at the other band and the complexity index are inferior to AUCs in the case using a single complexity index.

**Table 4:** The area under the ROC curve (AUC) for *c*_1_, |*c*_2_|, and sample entropy averaged scale 1–5.

	**AUC (SD)**
*c*_1_	0.862 (0.029)
|*c*_2_|	0.857 (0.026)
sample entropy	0.850 (0.026)

*In this case, c_1_, |c_2_|, and sample entropy, each first-third principal components, were used separately. Here, AUC values were averaged among 20 trials to choose tested and evaluated data set in 5-fold cross-validation and their standard deviations (SD) were also derived*.

**Table 5:** AUC for the NS of the PLI. In this case, the NS of the PLI was used separately for each of the first-third principal components.

	**AUC (SD)**
NS at delta	0.600 (0.055)
NS at theta	0.545 (0.053)
NS at alpha	0.840 (0.030)
NS at beta	0.785 (0.034)
NS at gamma	0.708 (0.058)

*Here, AUC values were averaged among 20 trials to choose tested and evaluated data set in 5-fold cross-validation and their standard deviations (SD) were also derived*.

**Table 6:** AUC for the combination of *c*_1_ and |*c*_2_| and the combination of the NS at alpha, *c*_1_, |*c*_2_| and sample entropy.

	**AUC (SD)**	**t-value (p-value) with *c*_1_**	**t-value (p-value) with |*c*_2_|**	**t-value (p-value) with sample entropy**	**t-value (p-value) with NS at alpha**
*c*_1_ & |*c*_2_|	0.885 (0.038)	***t*** **= 2.69 (*****p*** **= 0.014)**	***t*** **= 2.96 (*****p*** **= 0.007)**	-	-
*c*_1_ & NS at alpha	0.881 (0.046)	*t* = 1.82 (*p* = 0.083)	-	-	***t*** **= 4.82 (p <0.001)**
|*c*_2_| & NS at alpha	0.887 (0.035)	-	***t*** **= 3.31 (*****p*** **= 0.003)**	-	***t*** **= 5.79 (*****p*** ** <0.001)**
sample entropy & NS at alpha	0.873 (0.031)	-	-	***t*** **= 3.27 (*****p*** **= 0.004)**	***t*** **= 4.37 (*****p*** **< 0.001)**

*In this case, the first-third principal components were used separately. Here, AUC values were averaged among 20 trials to choose tested and evaluated data set in five–fold cross-validation and their standard deviations (SD) were also derived. The paired t-value comparing with AUC only used single measure (c_1_, |c_2_|, sample entropy.) The t and corresponding p values with p < 0.05 are represented by bold characters. Positive t value indicates the increased AUC in the combination case*.

**Figure 8:**
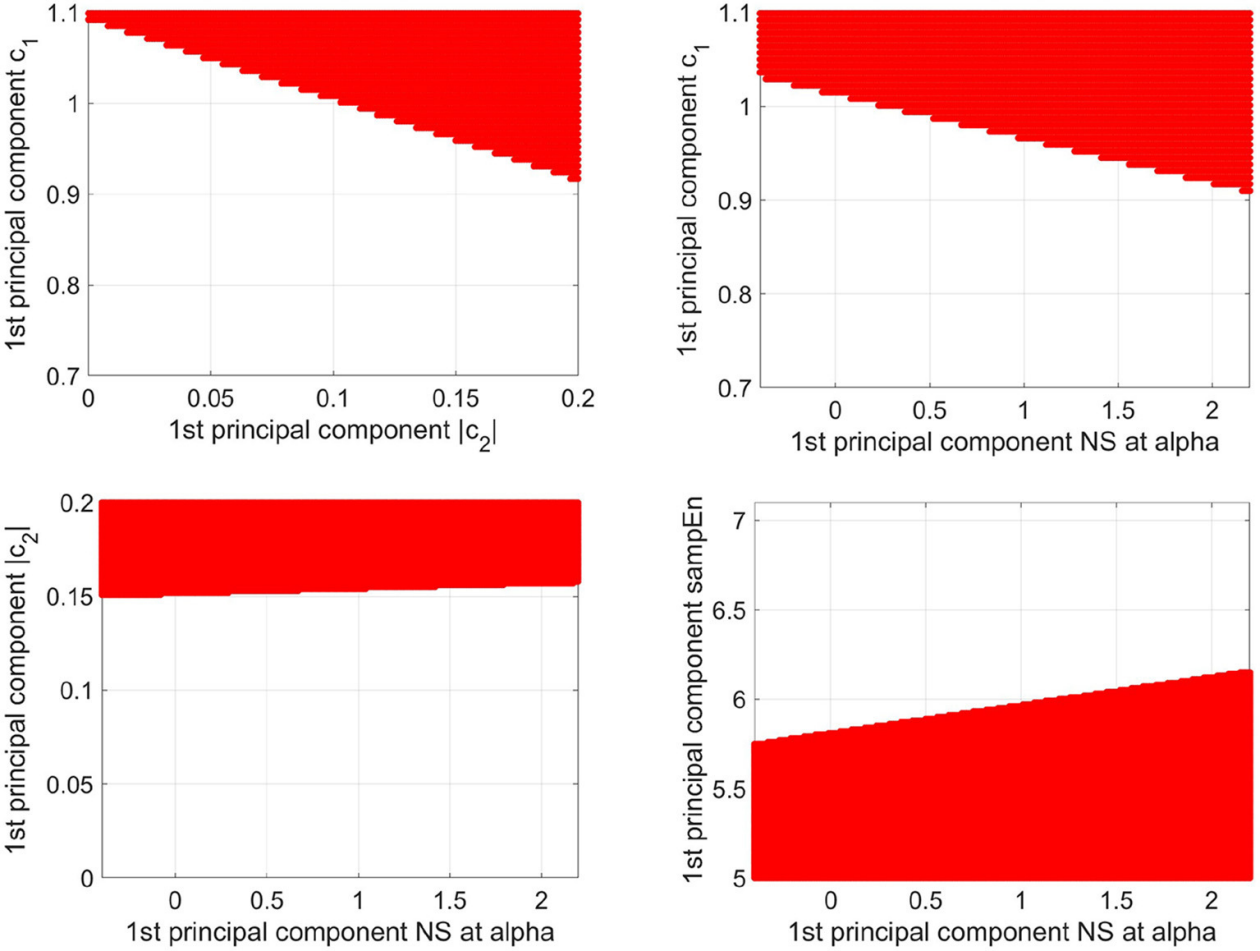
Decision region (represented as the red region) for older participants with a decision probability of more than 0.9, obtained via logistic regression, is shown on the plane between the 1st principal component of *c*_1_ and the 1st principal component of |*c*_2_| (upper left part), the plane between the 1st principal component of *c*_1_ and the 1st principal component of NS at alpha (upper right part), and the plane between the 1st principal component of |*c*_2_| and the 1st principal component of NS at alpha (lower left part), and the plane between the 1st principal component of sample entropy and the 1st principal component of the NS at alpha (lower right part). Here, the other components, except for the axis of the planes, were set to average among participants in both the younger and older groups. The dependency on all these factors in the decision region was confirmed.

To demonstrate that the determination area for older participants is determined by *c*_1_, |*c*_2_|, sample entropy, and the NS at alpha of the PLI, the determination area of the older participants was defined as the plane between the first principal components of *c*_1_ and first principal components of |*c*_2_|, and plane between the first principal components of *c*_1_ and first principal components of the NS at alpha, plane between the first principal components of |*c*_2_| and first principal components of the NS at alpha, and plane between the first principal components of sample entropy and first principal components of the NS at alpha (see Figure 8). Here, the other components, with the exception of the axis of the planes, were set to average among participants in both the younger and older study groups. The dependency on all of these factors in the decision region was confirmed.

## 4. Discussion

In this study, we investigated the relationship between complexity and functional connectivity in aging via EEG. In the MF analysis, we found that *c*_1_ (as the index for the smoothness of the EEG signal) decreased with aging, and |*c*_2_| (which is an index of a multi-fractal nature) also decreased with aging. In the MSE analysis, a statistically significant region-specific increase in the small-temporal-scale sample entropy of aging was observed. In the PLI analysis, we found that functional connectivity increased in the delta and gamma bands with aging. In the comparison of the classification accuracy among *c*_1_, |*c*_2_|, small-temporal-scale sample entropy, and the NS of the PLI, *c*_1_ demonstrated the highest classification accuracy (AUC = 0.86). Considering the complementary relationship between complexity and functional connectivity, the accuracy of aging classification improved based on the current study results.

It is imperative to discuss the reason why *c*_1_, which is an index of smoothness, decreases with aging. Gamma activity has been reported to increase with age (Böttger et al., 2002). In our results, a higher relative power of the gamma band in the major part of the electrodes was confirmed (see Supplementary Material). A previous study demonstrated that the degree of complexity of EEG signals predominantly depends on smaller temporal scale (i.e., fast frequency component) behaviors, instead of on larger temporal scale (i.e., slow frequency component) behaviors (Nobukawa et al., 2019b). Our results exhibit a tendency corresponding with these findings; that is, it can be interpreted that our observed increasing complexity (decreasing *c*_1_ and increasing smaller temporal-scale sample entropy) is induced by increasing gamma activity due to aging.

In the gamma band, artifacts due to muscle activity are larger compared to lower frequency bands (Whitham et al., 2007, 2008). Therefore, although the majority of the time segments with artifacts involving muscle activity were removed in the evaluation epochs, it was essential to investigate the influence of muscle activity on gamma band results in the estimation of functional connectivity and complexity. Table 7 shows the results of younger vs. older groups repeated-measures ANOVA with the mean values of relative power at the gamma band among 16 electrodes as covariate in *c*_1_, |*c*_2_|, mean values of sample entropy in low temporal-scale regions 1 to 5 (0.005–0.025 s), and NS at gamma band. Resultingly, the group difference is maintained in *c*_1_, |*c*_2_|, and small-temporal-scale sample entropy. However, the group difference was not confirmed in the NS at gamma band. Therefore, in an epoch involving muscle activity, functional connectivity at the gamma band might be more strongly affected.

**Table 7:** Younger vs. older repeated–measures ANOVA results [*F* value (*p* value, partial η^2^)] with mean values of relative power at gamma band among 16 electrodes as covariate in *c*_1_, |*c*_2_|, mean values of sample entropy in low temporal-scale regions 1 to 5 (0.005–0.025 s), and NS at gamma band.

	**Group**	**Group × node**	**degree of freedom (ϵ)**
*c*_1_	***F* = 9.059 (*p* = 0.004, η^2^ = 0.162)**	*F* = 1.109 (*p* = 0.356, η^2^ = 0.023)	5.151 (ϵ = 0.343)
|*c*_2_|	*F* = 4.143 (*p* = 0.047, η^2^ = 0.081)	*F* = 1.009 (*p* = 0.420, η^2^ = 0.021)	6.023 (ϵ = 0.402)
sample entropy	***F* = 10.179 (*p* = 0.003, η^2^ = 0.178**)	*F* = 1.104 (*p* = 0.355, η^2^ = 0.023)	3.792 (ϵ = 0.253)
NS at gamma	*F* = 0.127 (*p* = 0.723, η^2^ = 0.003)	*F* = 1.704 (*p* = 0.109, η^2^ = 0.035)	6.853 (ϵ = 0.457)

*F and p values with p < 0.05 are represented by bold characters. Degree of freedom and Greenhouse-Geisser adjustments ϵ in the interaction for group × node are also shown*.

Moreover, we must consider the reason why the relationship between multi-fractality and complexity and their underlying neurophysiological mechanism. The time-series with large (small) multi-fractality exhibits intermittent and transient behavior with large (small) amplitude (Ihlen, 2012). Complexity reflects the degree of complexity for temporal behavior in the entire time-range, instead of intermittent behavior. Therefore, EEG signal in older subjects corresponds to homogeneous and high complex temporal behaviors. In the aging process, the connectivity of the wide range of inter neural networks becomes weak and the neural noise increases; consequently, the amount of network communication decreases (Cremer and Zeef, 1987; Onoda et al., 2012; Nobukawa et al., 2019a). Therefore, the amplitude of intermittent transient behavior driven by the neural activities from the other regions might become weak, that is, the decreased multi-fractality might reflect fewer global neural interactions. Regarding complexity, as mentioned above, increasing complexity (decreasing *c*_1_ and increasing smaller temporal-scale sample entropy) is induced by increasing gamma activity due to aging. Considering fact that gamma-band activity relates to local excitatory and inhibitory neural interaction (Börgers and Kopell, 2003), increasing complexity is caused by the alternation of local regional neural activity.

Furthermore, it is necessary to consider why the classification accuracy is improved by adding the NS at alpha. The activity of the neural network alternates region-specifically with aging [as reviewed in Reuter-Lorenz (2002)]. In our result, NS at alpha exhibits significant high region-specificity (see Table 3). Such age-related region-specific characteristics could be extracted by principal component analysis and logistic regression; consequently, a relatively high classification accuracy was thus obtained in the current study. Furthermore, in recent years, studies on complexity and functional coupling have pointed out complementary relationships (Ghanbari et al., 2015; Nobukawa et al., 2020a); the studies have reported that their combination improves the detection accuracy of pathological conditions. This relationship is attributed that the inter-regional neural interactions as functional connectivities induce the local regional variability (Sporns et al., 2007; Misic et al., 2011), which was observed between NS and multi-fractality |*c*_2_| (see Figures 6, 7). The complementarity was also observed in the decision plan for this study (see Figure 8). Based on these results, we conclude that the combination of complexity and the PLI likely improves the classification accuracy of aging.

Finally, in addition to the substantial strengths of the current investigation, the limitations of this study need to be considered. The EEG signal does not always reflect the neural activity just below the electrodes. In this study, EEG was measured using 16 electrodes, which is less than the current number of electrodes recommended by the International Federation of Clinical Neurophysiology (Seeck et al., 2017). Therefore, using MEG and high dense EEG with increased high spatial resolution and applying cortical positioning method might enhance the ability to identify the complex functional connection structures caused by aging. Regarding temporal-scale resolution, recently, Kosciessa et al. (2020) indicated an issue in the coarse-grain process's ability to rigidly extract the complexity with temporal-scale specificity (Kosciessa et al., 2020). Since the age-related alternation of power was distributed in wide frequency bands in this study, the need for a more appropriate method to extract temporal-scale dynamics is important to thoroughly investigate neural interactions and temporal-scale specific complexity. In future studies, these points should be dealt with.

## 5. Conclusion

In this study, we were able to portray the changes in neural activity with aging by using MF and MSE analyses, which are complexity analyses, as well as PLI analysis, which evaluates the functional connections. Classification accuracy was improved by combining functional connectivity, which has a complementary relationship with the index of complexity. Despite certain limitations, the outcome of this study demonstrates that the complementary relationship between complexity and functional connectivity within EEG plays an important role in detecting age-related changes in neural activity. Therefore, these results could be useful in formulating interventions for the prevention of age-related brain dysfunction.

## Data Availability Statement

The datasets presented in this article are not readily available because the informed consent did not include the declaration regarding publication of clinical data. Requests to access the datasets should be directed to Sou Nobukawa, nobukawa@cs.it-chiba.ac.jp.

## Ethics Statement

The studies involving human participants were reviewed and approved by the Ethics Committee of Kanazawa University. The patients/participants provided their written informed consent to participate in this study.

## Author Contributions

MA, SN, MK, and TT designed the methods. MA and SN analyzed the results, wrote the main manuscript text, and prepared all the figures. MK conducted the experiments. All authors reviewed the manuscript.

## Funding

This work was supported by JSPS KAKENHI for Early-Career Scientists (grant number 18K18124) (SN), Grant-in-Aid for Scientific Research (C) (grant number 20K07964) (TT), and The Okawa Foundation for Information and Telecommunications (grant number 20–20) (SN).

## Conflict of Interest

The authors declare that the research was conducted in the absence of any commercial or financial relationships that could be construed as a potential conflict of interest.

## Publisher's Note

All claims expressed in this article are solely those of the authors and do not necessarily represent those of their affiliated organizations, or those of the publisher, the editors and the reviewers. Any product that may be evaluated in this article, or claim that may be made by its manufacturer, is not guaranteed or endorsed by the publisher.
