# Supplementary material for: Alteration of Neural Network Activity With Aging Focusing on Temporal Complexity and Functional Connectivity Within Electroencephalography
Source: Front Aging Neurosci. 2022 Feb 4;14:793298. doi: 10.3389/fnagi.2022.793298 (PMC8855040; doi:10.3389/fnagi.2022.793298)
Supplement: Supplementary file 1 [file Data_Sheet_1.PDF]

Supplementary Material

1 RELATIVE POWER ANALYSIS

A relative power analysis was performed supplementary to the research results. Table S1 shows the repeated–measures analysis of variance (ANOVA) results for the relative power for each band between the younger and older participants. Statistically significant large group effects at the alpha, beta, and gamma bands and statistically significant group $\times$ node interactions at the theta, alpha, beta, and gamma bands were confirmed. Post-hoc t–test results for the relative power are shown in the lower part of Fig. S1. Figure S1 shows the mean value of the relative power in the younger and older groups, as well as the post-hoc t–test results evaluating the relative power. In the alpha band, a statistically significant lower relative power for the older group was confirmed at all electrodes except for Fp1. In the beta band, a statistically significant higher relative power was confirmed at all electrodes except for Fp1. In the gamma band, a statistically significant higher relative power was confirmed at the Fp2, F3, F4, F8, Fz, C3, C4, P4, Pz, O1, and O2 electrodes and a lower relative power was confirmed at the C3 electrode.

2 EXAMPLES OF MULTIFRACTAL TIME-SERIES

For the monofractal time series, singular value spectrum $D(h)$ converges to its 1st cumulant c_1, which is the Hölder index h value at moment $q = 0$. For the multi-fractal time series, $D(h)$ is distributed around c_1. Therefore, the degree of distribution of $D(h)$ reflects the multi-fractal property, which corresponds to absolute value of 2nd cumulant $|c_2|$. The time-series with large (small) multi-fractality ($|c_2|$) exhibits intermittent and transient behavior with large (small) amplitude. The complexity notified by c_1 reflects the degree of complexity for temporal behavior in entire time–range, instead of intermittent behavior. Figure S2 shows the time–series of exponential-translated Blown motion: $\exp(\alpha(W(t)+2.5)$ where α and $W(t)$ represent amplitude of instantaneous transient behavior and Blown motion ($\alpha = 0.5, 1.5, 2.5, 3.5$), respectively, and the corresponding $D(h)$ ($-5 \leq q \leq 5$). With increasing amplitude of instantaneous transient behavior, the range of $D(h)$ increases, i.e., $|c_2|$ increases.

Figure S3 shows the results of multifractal analysis for one younger participant. c_1 indicates the value of h when $D(h) = 1.0$ ($q = 0$), and the absolute value of c_2 corresponds to the range of the $D(h)$ distribution between $q = -5$ and $q = 5$.

Table S1. Repeated–measures analysis of variance (ANOVA) power analysis results (F value (p value, partial η^2)) between the younger and older participants; F and p values with $p < 0.05$ are represented by bold characters. Age group was used as an inter-subject factor, and the 16 electrodes from Fp1 to T6 were used as intra-subject factors. Degree of freedom and Greenhouse-Geisser adjustments ϵ in the interaction for group $\times$ nodes are also shown.

	Group	Group $\times$ nodes	Degree of freedom (ϵ)
delta band	$F = 1.092$ ($p = 0.301, \eta^2 = 0.022$)	$F = 0.721$ ($p = 0.523, \eta^2 = 0.015$)	2.613 ($\epsilon = 0.174$)
theta band	$F = 0.001$ ($p = 0.974, \eta^2 < 0.001$)	$\boldsymbol{F = 4.493}$ ($\boldsymbol{p < 0.001, \eta^2 = 0.086}$)	4.559 ($\epsilon = 0.304$)
alpha band	$\boldsymbol{F = 23.777}$ ($\boldsymbol{p < 0.001, \eta^2 = 0.331}$)	$\boldsymbol{F = 4.458}$ ($\boldsymbol{p = 0.004, \eta^2 = 0.085}$)	3.298 ($\epsilon = 0.220$)
beta band	$\boldsymbol{F = 45.847}$ ($\boldsymbol{p < 0.001, \eta^2 = 0.489}$)	$\boldsymbol{F = 8.185}$ ($\boldsymbol{p < 0.001, \eta^2 = 0.146}$)	4.219 ($\epsilon = 0.281$)
gamma band	$\boldsymbol{F = 18.125}$ ($\boldsymbol{p < 0.001, \eta^2 = 0.274}$)	$\boldsymbol{F = 2.834}$ ($\boldsymbol{p = 0.03, \eta^2 = 0.056}$)	3.679 ($\epsilon = 0.245$)

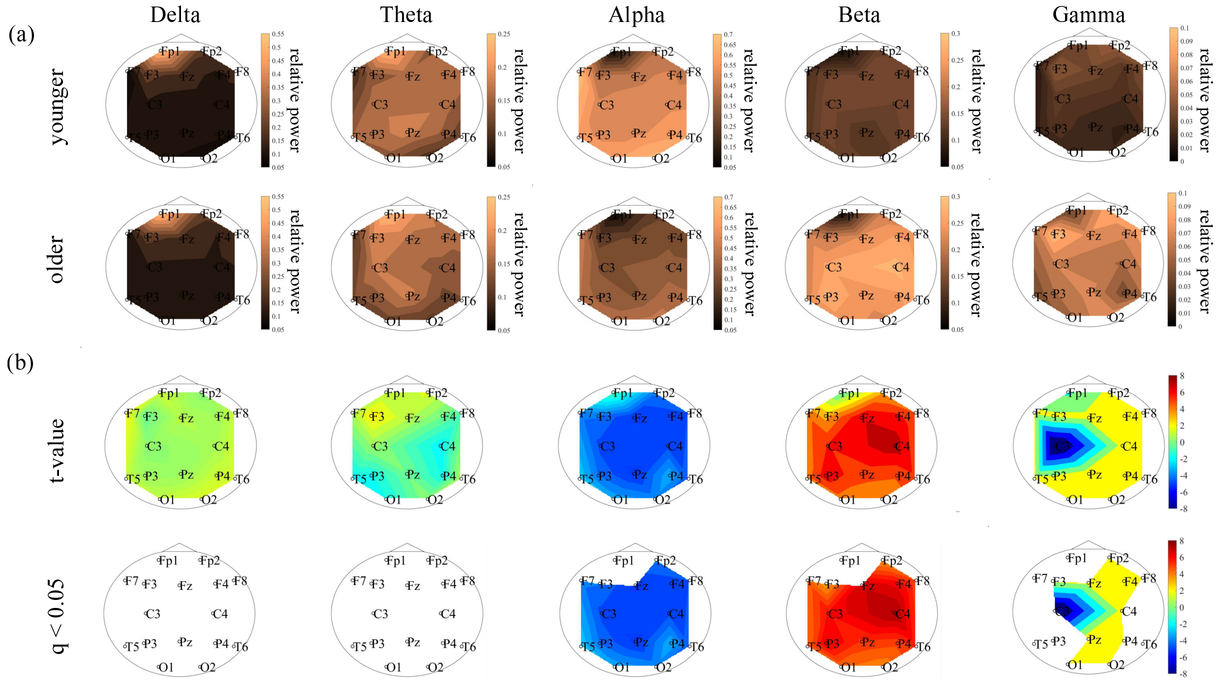

Figure S1: (a) The mean values for the relative power at the delta, theta, alpha, beta, and gamma bands in the younger (upper parts) and older (lower parts) groups. (b) t-values between the younger and older groups (upper parts). The warm (cold) colors represent higher (smaller) power values in the older versus the younger groups. t–values satisfying the false discovery rate (FDR) correction criterion of $q < 0.050$ (lower parts).

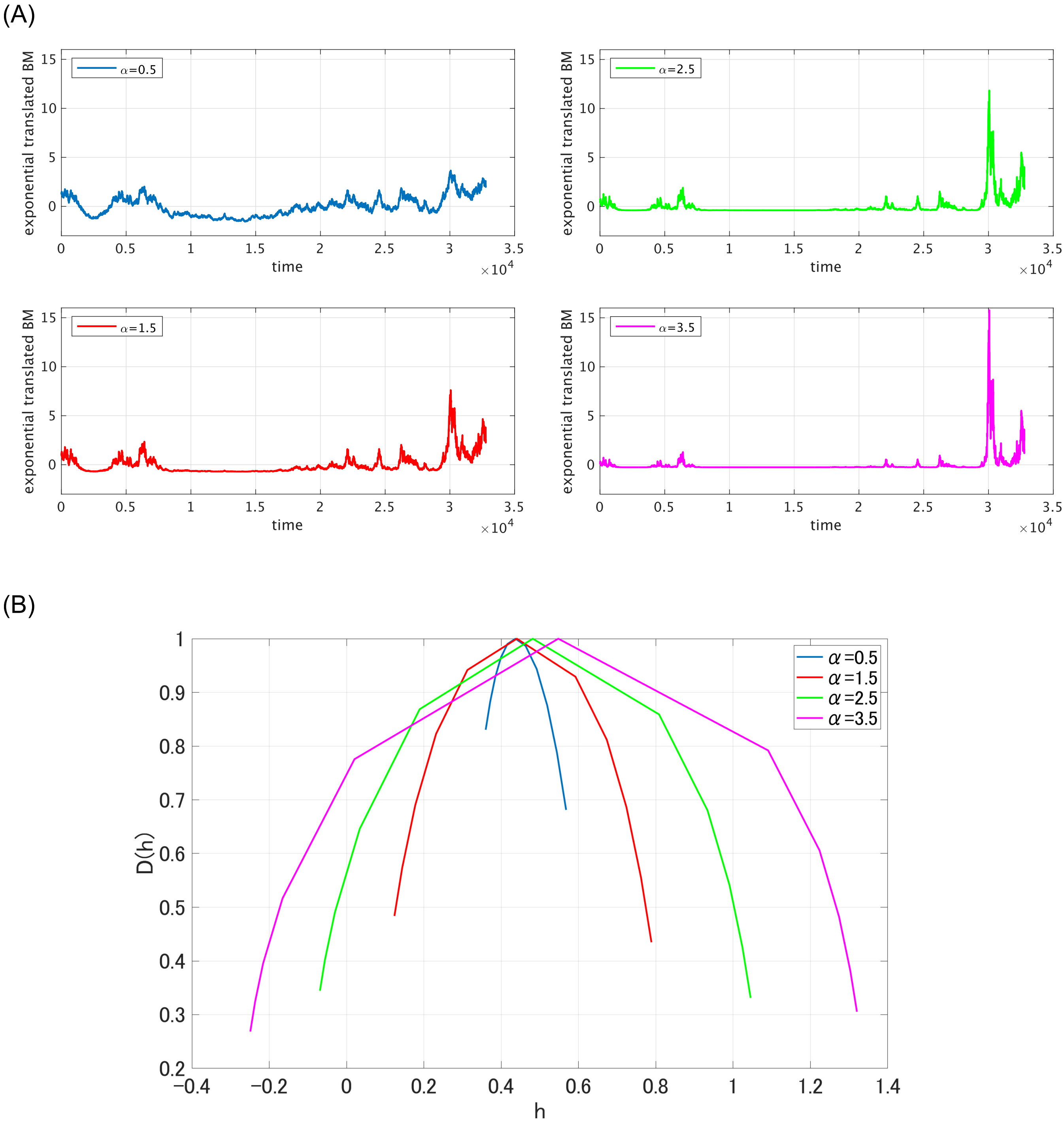

Figure S2: (A) Time-series of exponential-translated Blown motion: $\exp(\alpha(W(t) + 2.5)$ where $alpha$ and $W(t)$ represent amplitude of instantaneous transient behavior and Blown motion $(alpha = 0.5, 1.5, 2.5, 3.5)$, respectively. (B) Corresponding $D(h)$ $(-5 \leq q \leq 5)$. With increasing amplitude of instantaneous transient behavior, the range of $D(h)$ increases, i.e., $|c_2|$ increases.

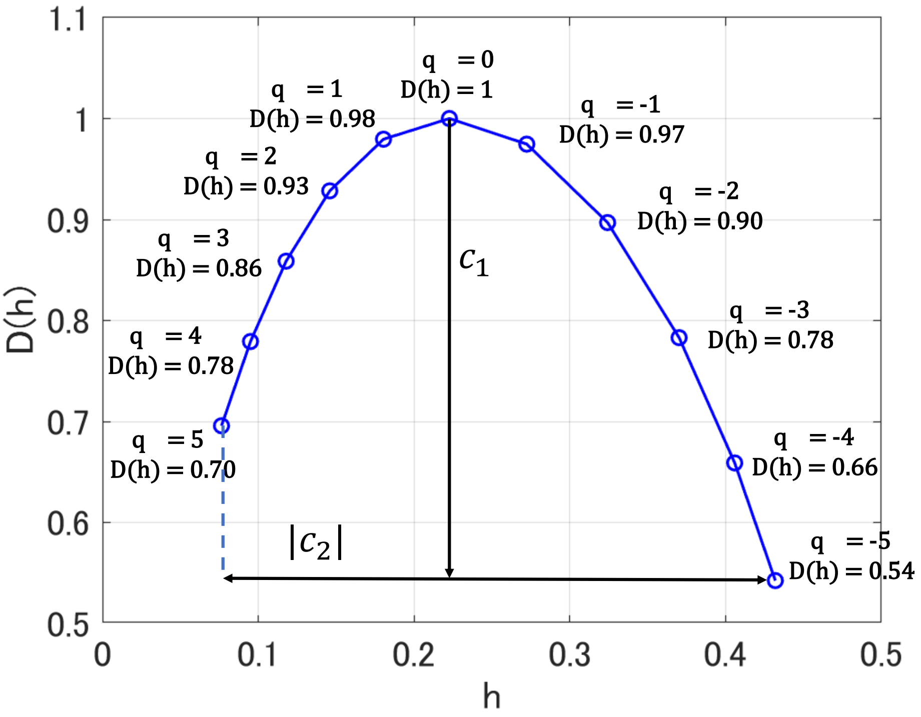

Figure S3: Singular value spectrum $D(h)$ in multifractal analysis for one younger subject. Here, h represents the Hölder exponent. c_1 shows the h value, where $D(h) = 1.0$ ($q = 0$) and the absolute value of c_2 correspond to the range of the $D(h)$ distribution between $q = -5$ and 5.
